# How haptophytes microalgae mitigate vitamin B_12_ limitation

**DOI:** 10.1038/s41598-019-44797-w

**Published:** 2019-06-10

**Authors:** Charlotte Nef, Sébastien Jung, Francis Mairet, Raymond Kaas, Dominique Grizeau, Matthieu Garnier

**Affiliations:** 1 IFREMER, Physiology and Biotechnology of Algae Laboratory, rue de l’Ile d’Yeu, 44311 Nantes, France; 2 Université de Nantes, CNRS, GEPEA, 44602 Saint-Nazaire, France

**Keywords:** Ecophysiology, Microbial ecology

## Abstract

Vitamin B_12_ (cobalamin) can control phytoplankton development and community composition, with around half of microalgal species requiring this vitamin for growth. B_12_ dependency is determined by the absence of cobalamin-independent methionine synthase and is unrelated across lineages. Despite their important role in carbon and sulphur biogeochemistry, little is known about haptophytes utilization of vitamin B_12_ and their ability to cope with its limitation. Here we report the first evaluation of B_12_ auxotrophy among this lineage based on molecular data of 19 species from 9 families. We assume that all species encode only a B_12_-dependent methionine synthase, suggesting ubiquitous B_12_ auxotrophy in this phylum. We further address the effect of different B_12_ limitations on the molecular physiology of the model haptophyte *Tisochrysis lutea*. By coupling growth assays in batch and chemostat to cobalamin quantification and expression analyses, we propose that haptophytes use three strategies to cope with B_12_ limitation. Haptophytes may assimilate dissolved methionine, finely regulate genes involved in methionine cycle and B_12_ transport and/or limit B_12_ transport to the mitochondrion. Taken together, these results provide better understanding of B_12_ metabolism in haptophytes and represent valuable data for deciphering how B_12_-producing bacteria shape the structure and dynamics of this important phytoplankton community.

## Introduction

Vitamin B_12_, or cobalamin, can control phytoplankton growth^1^ and community composition in Polar Regions^2–4^ including the Southern Ocean^5,6^, and in some temperate coastal waters^7^. This organometallic cobalt-containing cofactor is only produced by certain species of archaea and bacteria. Cobalamin biosynthesis involves 30 enzymatic steps^8–10^ and eukaryotes, including algae, do not have the complete genetic equipment^11,12^. The metabolic need for cobalamin is relatively common among microalgae, with around 50% species being B_12_-auxotrophic^11–13^. Therefore, either through direct interactions^11,14^ or by cell lysis and release^15^, prokaryotes are the ultimate source of vitamin B_12_ for auxotrophic primary producers. Among phytoplankton species, haptophytes, whose origin has been dated around 830 million years ago^16^, are important contributors to global marine primary production, representing significant carbon sink in oceans^17,18^. These widespread eukaryotic microalgae are also one of the main producers of dimethylsulfoniopropionate (DMSP), the precursor of dimethyl sulfide (DMS), an important component of sulphur cycle that acts as a cloud condensation nuclei^19,20^. Thus, understanding how haptophytes acclimate to cobalamin limitation appears relevant for elucidating primary production and nutrient cycling processes in oceans.

**Figure 1:**
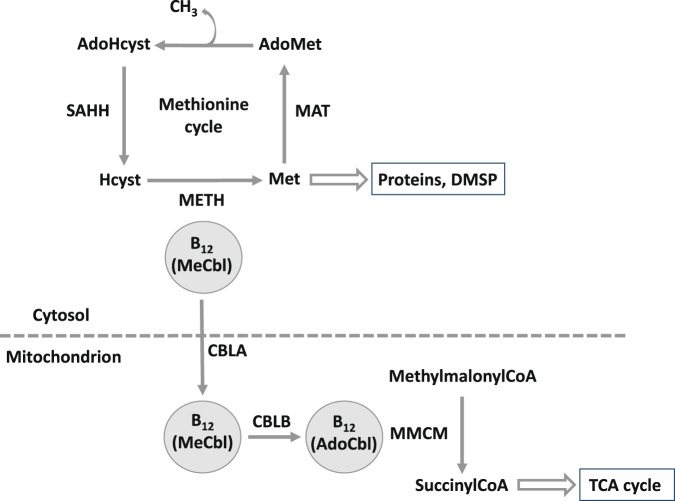
Schematic diagram of B_12_ utilization in eukaryotic C1 metabolism. B_12_ active forms methylcobalamine (MeCbl) and adenosylcobalamin (AdoCbl) catalyze different enzymatic reactions. B_12_-dependent METH uses MeCbl in the cytosol and B_12_-requiring MMCM needs AdoCbl in the mitochondrion. AdoHcyst, S-adenosylhomocysteine; AdoMet, S-adenosylmethionine; Hcyst, homocysteine; Met, methionine; TCA cycle, tricarboxylic acid cycle.

Within eukaryotes, vitamin B_12_ enables the activity of a relatively few number of enzymes: methionine synthase, class II ribonucleotide reductase (RNR II) and methylmalonyl-CoA-mutase (MMCM). Cobalamin has two active forms, methylcobalamin (MeCbl) and adenosylcobalamin (AdoCbl), permitting the activity of different enzymes. Methionine synthases are key enzymes for the production of proteins as they allow the conversion of 5-methyltetrahydrofolate and homocysteine into tetrahydrofolate and methionine. Whereas the first isoform of methionine synthase (METH, gene *metH*) needs MeCbl as cofactor and is encoded in all microalgae, the second isoform (METE, gene *metE*) does not need cobalamin, has a lower catalytic rate^21^ and is found in B_12_-independent species^11,12,22^. RNR II converts ribonucleotides into deoxyribonucleotides for DNA synthesis using MeCbl^9^ and MMCM (gene *mmcm*) is involved in the citric acid (TCA) cycle in the mitochondrion, where it converts methylmalonyl-CoA into succinyl-CoA with AdoCbl^13^. Nonetheless, species with these B_12_-dependent enzymes can grow without the vitamin if they possess the cobalamin-independent METE isoform. This suggests that B_12_-dependent reactions other than methionine synthesis are less critical for their development in cobalamin-deprived environments. In addition, accessory proteins CBLA and CBLB allow B_12_ transport of MeCbl and conversion into AdoCbl in the mitochondrion for MMCM activity^23^ (Fig. 1).

It has been proposed that loss of B_12_-independent methionine synthase arose multiple times in evolution^12,13,24^. A reason advanced would be that microalgae provided with a non-limiting supply of cobalamin would lost selective pressure on the energy-expensive^25^ METE and retain only METH. As an example, a recent work on *Chlamydomonas reinhardtii* grown with a source of B_12_ revealed a shift from cobalamin-independence to auxotrophy^24^. The conversion of methionine from homocysteine is essential in one-carbon metabolism as methionine undergoes several ways of use^26^. It is either assimilated into proteins, or converted by the enzyme methionine adenosyltransferase (MAT, gene *metK*) into S-adenosylmethionine (SAM), an important methyl donor and radical source^22,27^ (Fig. 1). There are many reactions involving SAM demethylation, such as DNA methylation, synthesis of vitamin B_1_ (thiamine)^22,28^ and DMSP biosynthesis^29^. SAM demethylation leads to the formation of S-adenosylhomocysteine (SAH) which is finally hydrolyzed to regenerate homocysteine by the S-adenosylhomocysteine hydrolase (SAHH, gene *sahH*). All these reactions from methionine production to homocysteine regeneration are described as the methionine cycle (Fig. 1).

Interestingly, the only way known for marine microalgae to produce DMSP implies both SAM demethylation and methionine transamination, which suggests that DMSP synthesis is an important sink of methionine^29^. The majority of DMSP production in the ocean is due to haptophytes and dinoflagellates and, as this molecule does not contain nitrogen, it is suggested that it acts in microalgae as a dissipating excess energy agent when sulphur assimilation exceeds nitrogen incorporation^29^. Numerous species from these lineages are considered to be cobalamin-dependent ^11,13^. Therefore, vitamin B_12_ may be particularly important in haptophytes and dinoflagellates cellular processes, especially in nitrogen-limited environments.

Previous studies based on culture assays showed that on the 22 haptophytes species tested, 8 were able to grow without B_12_ addition and were considered as B_12_-independent^11,13^. In absence of culture assay in truthful axenic condition and of molecular evidence for the presence of METE in these species, the cobalamin dependence of haptophytes lineage stays unclear. Moreover, the question of how haptophytes acclimate and regulate key metabolic enzymes in B_12_ limitation stays poorly documented. Considering that haptophytes are major contributors to nano and pico-plankton communities^17,30^ and play a significant role in organic matter cycling, deciphering B_12_ dependence and B_12_-associated metabolism of this lineage is of global importance.

Here, our analysis of genes *metH* and *metE* of 19 genome-sequenced or transcriptome-sequenced haptophyte species suggest that the auxotrophy for B_12_ is ubiquitous in the haptophyte lineage. In a second part, by using batch and continuous cultures in controlled photobioreactors, we investigated the effect of different levels of B_12_ limitation on the molecular physiology of the model haptophyte *Tisochrysis lutea*. Genes expression analyses showed that methionine cycle is finely regulated by B_12_ availability in the environment.

## Results

### Phylogenetic analysis of methionine synthase in haptophytes

**Figure 2:**
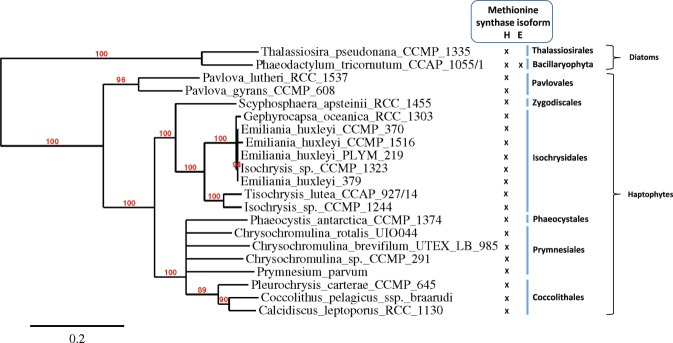
Phylogenetic tree of haptophytes inferred from B_12_-dependent methionine synthase (METH) using maximum likelihood. Bootstraps values are indicated at the nodes with values above 80% shown. Crosses indicate presence of either METH or METE in the dataset. Haptophyte and diatoms orders are indicated.

A survey of methionine synthase isoforms in 19 haptophyte species based on transcriptomic and genomic datasets has been conducted (see Supplementary Table 1 on Supplementary Information for sequences details). All samples investigated contained the B_12_-dependent *metH*. The phylogeny of this phylum was reconstructed from this gene, with species from orders Coccolithales, Isochrysidales and Prymnesiales relevantly gathered (Fig. 2). This reconstruction was consistent with what is usually found for 18S sequence^31^, suggesting an absence of horizontal gene transfer. The cobalamin-independent isoform *metE* was not found in any of the samples, indicating that all 19 species are B_12_-auxotrophic (Fig. 2). The Marine Atlas of Tara Ocean Unigenes (MATOU) for eukaryotic data^32^ was also investigated by searching similar genomic and proteomic sequences of METE from *Phaeodactylum tricornutum* and *Chlamydomonas reinhardtii*. The MATOU database gather large-scale environmental metatranscriptomic and metagenomic information. Since no haptophyte sequence was retrieved in these large datasets, this reinforces the hypothesis of absence of B_12_-independent methionine synthase in the haptophyte lineage.

Considering that comprehensive genomic and transcriptomic data of *Tisochrysis lutea* (Isochrysidaceae) were available, it was taken as model species for molecular physiology analyses depending on cobalamin quotas. *In silico* searches in *T*. *lutea* (strain CCAP 927/14) genome^31,33^ allowed to identify several genes involved in vitamin B_12_ metabolism, conversion and transport. Gene *metH* coding for cobalamin-dependent methionine synthase was found and the presence of the related protein was confirmed in our proteomic dataset. The B_12_-independent methionine synthase was not found in our proteomic nor genomic data, suggesting B_12_ auxotrophy. Translated sequences of METH protein from other haptophytes were compared with the one of *T*. *lutea* when possible (see Supplementary Table 1 of Supplementary Information).

### Assessment of B_12_ requirement of *Tisochrysis lutea*

**Figure 3:**
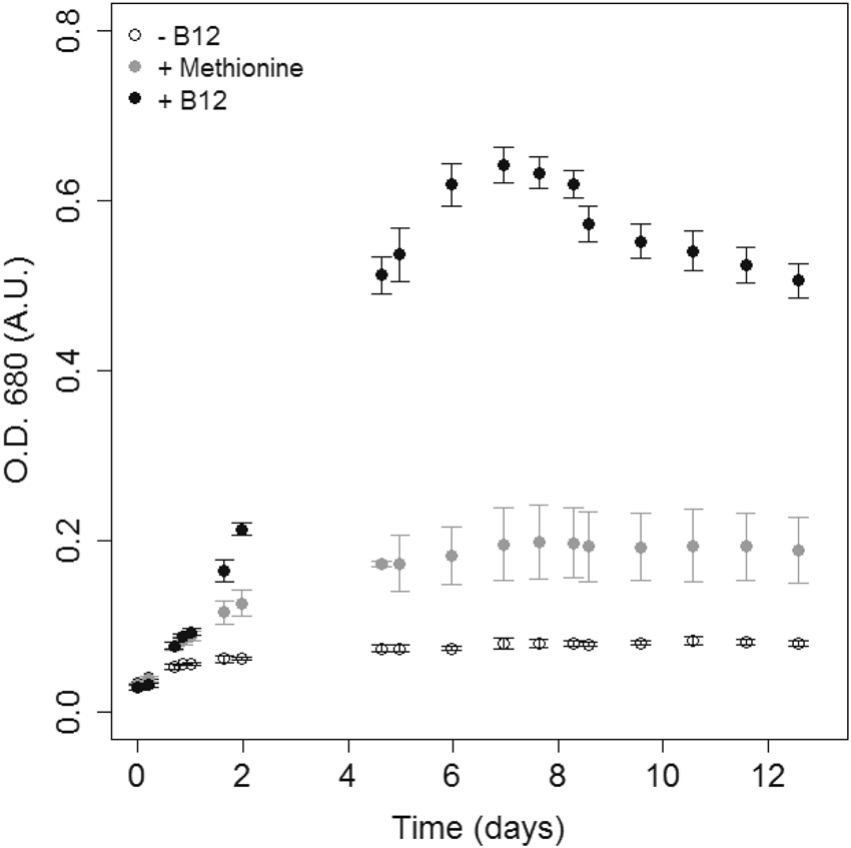
Growth curves of *Tisochrysis lutea* cultivated with either 40 ng L^−1^ vitamin B_12_, no vitamin B_12_ adding or 0.5 mg L^−1^ methionine. Values represent means of six biological replicates ± one standard deviation.

In order to validate biological dependency of *T*. *lutea* to vitamin B_12_, a growth assay was performed. The axenic microalgae were grown either in cobalamin-deprived medium, methionine adding or in complete medium. Cells grown with 40 ng L^−1^ cobalamin exhibited a maximal growth rate (*μ*_*max*_) of 0.35 ± 0.04 d^−1^ and a maximal biomass increase (Δ*C*_*max*_) of 0.48 ± 0.02 arbitrary units (A.U.) (Fig. 3). Cobalamin-free cultures showed a growth rate five times lower and statistically significant (*p* = 8.11 10^−8^; two-tailed Student’s *t* test) with *μ*_*max*_ = 0.07 ± 0.03 d^−1^ and Δ*C*_*max*_ = 0.05 ± 0.01 A.U. (Fig. 3). This was consistent with *in silico* analysis and clearly demonstrated *T*. *lutea* auxotrophy. The low growth observed for cobalamin-free cultures was due to the use of natural seawater which provided the cells with little naturally-present vitamin B_12_. Interestingly, microalgae grown with 0.50 mg L^−1^ methionine showed twice the growth of the negative control that was statistically significant (*p* = 4.04 10^−4^; two-tailed Student’s *t* test) with *μ*_*max*_ = 0.17 ± 0.04 d^−1^ and Δ*C*_*max*_ = 0.16 ± 0.04 A.U. (Fig. 3), meaning that *T*. *lutea* is able to uptake and assimilate dissolved methionine and use it instead of cobalamin. The assimilation of dissolved free amino acids by marine microalgae is not well documented. This result confirmed that cobalamin is vital for methionine synthesis and that a lack of B_12_ may induce a lack of methionine.

### B_12_-limited batch experiment

**Figure 4:**
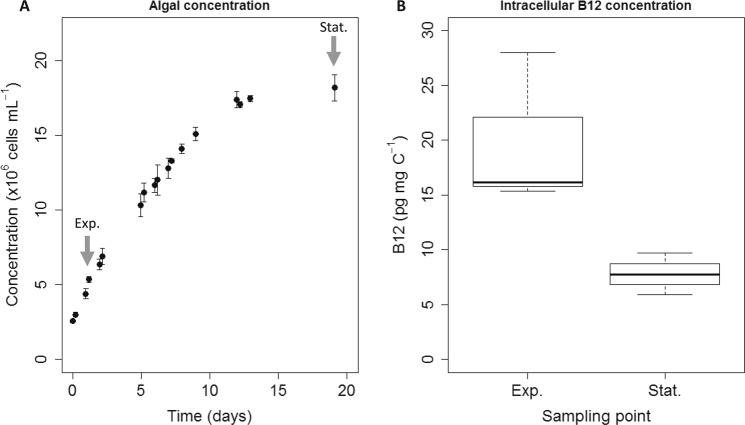
Batch cultures of *T*. *lutea* in B_12_-limited medium. (**A**) Growth curve of *T*. *lutea* (means of three biological replicates ± one standard deviation), with gray arrows indicating sampling points for vitamin B_12_ content and qPCR analysis. (**B**) Boxplot of intracellular cobalamin content at two sampling points during exponential (Exp.) and stationary (Stat.) phase with bold line indicating median (n = 3 replicates).

Cobalamin-limited batch culture in triplicate was set up to analyze expression of genes involved in vitamin B_12_ utilization, conversion and transport, and to compare their expression depending on cobalamin quota. Figure 4A presents the evolution of the average cell concentration against time and two sampling points for B_12_ and qPCR measurements. Figure 4B presents results for intracellular B_12_ measures, ranging from 20 ± 7 pg mg C^−1^ in early exponential phase to 8 ± 2 pg mg C^−1^ in late exponential phase, with a statistically significant two-fold decrease in intracellular cobalamin concentration due to vitamin starvation (*p* = 0.02; two-tailed Student’s *t* test). In their cobalamin-limited batch experiment, Cruz-Lopez *et al*.^34^ showed a two-fold decrease of B_12_ quota for the dinoflagellate *Lingulodinium polyedrum*^34^, which is consistent with our result.

**Figure 5:**
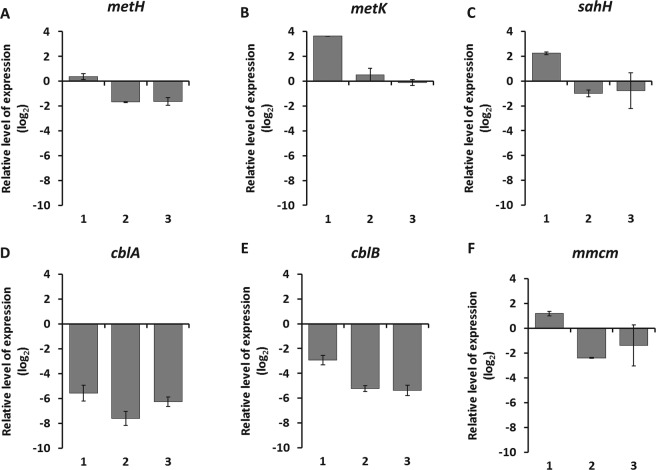
Genes expression in batch cultures of *T*. *lutea*. Relative levels of expression of (**A**) *METH*, (**B**) *METK*, (**C**) *SAHH*, (**D**) *CBLA*, (**E**) *CBLB* and (**F**) *MMCM* genes. Values represent expression level at stationary phase divided by expression level at early exponential phase. Data are log_2_ normalized. Values are shown for each biological triplicate (1, 2 and 3). Bars indicate means of technical triplicate measurements and error bars represent one standard deviation (see Table 2 on Supplementary Information for primers).

The expression of genes *metH*, *metK* and *sahH*, involved in the methionine cycle, *cblA*, *cblB* and *mmcm*, involved in cobalamin transport, conversion and utilization in the mitochrondrion was followed during high cobalamin availability (early exponential phase) and cobalamin starvation (stationary phase). The expression of methionine cycle genes and *mmcm* did not show a clear trend (Fig. 5A–C,F; Supplementary Dataset 1; Supplementary Fig. 2 in Supplementary Information). In comparison, genes *cblA* and *cblB* were significantly repressed (*p* = 0.02 and *p* = 0.04; two-tailed Student’s *t* test) by 72-fold and 11-fold respectively (Supplementary Dataset 1; Supplementary Fig. 2). This finding suggests that B_12_ starvation decreases expression of genes involved in cobalamin transport and conversion. It must be pointed out that growth rate decrease at the end of the batch culture may lead to cellular processes influencing many biochemical pools. Therefore, the expression of genes analyzed here may be the result of a global physiological state not specifically related to B_12_ starvation. A more accurate approach using cobalamin-limited chemostat was thus undertaken to confirm the effect of different vitamin B_12_ status on genes expression.

### Chemostat experiment in B_12_ limitation

#### Populations analysis

**Figure 6:**
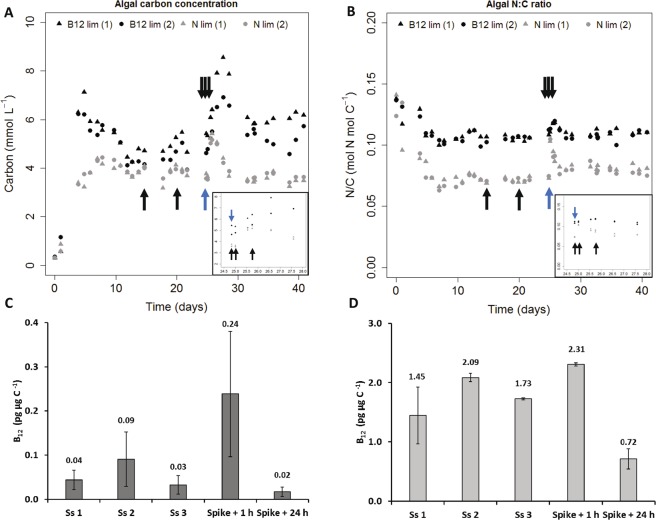
Chemostat cultures of *T*. *lutea*. (**A**) Algal carbon concentration and (**B**) algal N:C ratio. Blue arrows represent nutrient spike, black arrows indicate sampling points (Ss 1, 2, 3, spike + 1 h and spike + 24 h). (**C**,**D**) Intracellular cobalamin content at three sampling points during steady-state (Ss 1, 2, 3), 1 and 24 hours after nutrient input. Bars indicate values for the two biological replicates, with error bars representing the range. Data are for B_12_-limited (black) and nitrogen-limited (gray) chemostats.

To accurately describe the effect of vitamin B_12_ status on the expression of genes involved in cobalamin use, a B_12_-limited chemostat experiment was implemented in controlled photobioreactors. Nitrogen-limited (N-limited) chemostats with the same dilution rate were taken as controls to verify whether the observed results were specific to B_12_ limitation or rather related to a more general physiological status. Effort was made to prevent any bacterial contamination throughout the duration of the experiments. Particulate carbon and nitrogen and cellular concentration (see Supplementary Fig. 3 in Supplementary Information) were monitored at high frequency and allowed to accurately describe culture phases. Biological duplicates exhibited similar trends during all the duration of the experiment (Fig. 6A,B). Based on stability of carbon and microalgal concentration, steady-state was reached in both chemostats at day 12 (Fig. 6A,B; Supplementary Fig. 3). At day 25, a spike of limiting nutrient (vitamin B_12_ or nitrates) resulted in an increase in carbon biomass in all cultures, confirming nutrient limitation during the steady-state phase (Fig. 6A,B). Samples were collected on days 14, 21, 25, 26 and 27 for B_12_ content and qPCR analyses. For a same dilution rate, carbon content was slightly higher in B_12_ limitation than in N-limited control chemostats. Based on N/C results, physiological status of N-limited chemostats were described: nitrogen limitation at steady-state; nutrient repletion one hour after nitrogen input during N/C increase and nutrient depletion 24 hours after nitrogen input, at N/C decrease.

#### Intracellular B_12_ content

Intracellular cobalamin content was measured at different times in B_12_-limited chemostats and nitrogen-limited control cultures. Three samples were collected during steady-state. Mean cobalamin quota in B_12_-limited cultures prior to the cobalamin spike was 0.03 ± 0.02 pg μg C^−1^ (Fig. 6C). In comparison, B_12_ quota in N-limited chemostats was 1.73 ± 0.02 pg μg C^−1^, value 50 times greater than the one observed in B_12_-limited cultures (Fig. 6D). One hour after cobalamin spike, mean quota of B_12_-limited chemostats was multiplied by 8, reaching 0.24 ± 0.14 pg μg C^−1^, indicating an ability to quickly assimilate cobalamin (Fig. 6C). In N-limited cultures, B_12_ quota was on average nine times higher one hour after a spike of nitrogen (2.31 ± 0.03 pg μg C^−1^) relative to the one of B_12_-limited cultures after a cobalamin pulse (Fig. 6D). One day after nutrient spike, mean B_12_-limited chemostats quota dropped below steady-state value of 0.02 ± 0.01 pg μg C^−1^, suggesting rapid vitamin depletion (Fig. 6C), while after the nitrogen pulse the cobalamin quotas of the N-limited control cultures fell to 0.72 ± 0.17 pg μg C^−1^ (Fig. 6D), nearly 50 times higher than those of B_12_-limited cultures. This was likely attributable to the increase in cellular division, which was probably faster than vitamin acquisition. By combining B_12_ quotas and N/C ratio, physiological states for B_12_-limited chemostats were described: nutrient limitation at steady-state; nutrient repletion 1 hour after B_12_ input during N/C increase and nutrient depletion 24 hours after B_12_ input, at the end of N/C increase.

#### Molecular analyses

Genes expression analyses were carried out on samples collected at each physiological state to relate genes expression patterns to nutrient quotas. Genes for which expression was followed were the same as those of the batch experiment. Results presented for steady-state correspond to the third sample collected, just before nutrient spike.

**Figure 7:**
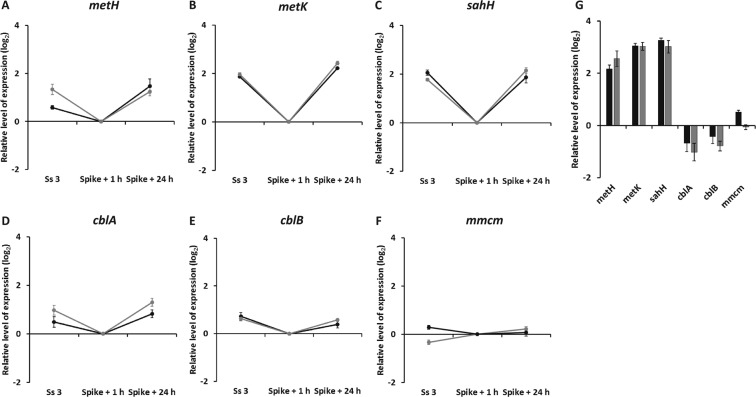
Genes expression in chemostat cultures of *T*. *lutea* during steady-state (Ss 3), 1 and 24 hours after nutrient spike: relative expression level of (**A**) *METH*, (**B**) *METK*, (**C**) *SAHH*, (**D**) *CBLA*, (**E**) *CBLB* and (**F**) *MMCM* genes normalized by mean expression level of cobalamin-limited chemostats at repletion, one hour after nutrient input; (**G**) barplot representing genes expression levels at Ss 3 in cobalamin-limited cultures normalized by their mean expression level in N-limited cultures at Ss 3. Data are log_2_ normalized. Points and bars indicate means of technical triplicates for each biological duplicate (represented in black and grey) and error bars represent one standard deviation (see Table 2 on Supplementary Information for primers).

As can be seen in Fig. 7G, at steady-state all genes involved in methionine cycle were more expressed in B_12_ limitation than in nitrogen limitation by 4-fold (*metH*) and 8-fold (*metK* and *sahH*) (Fig. 7G; Supplementary Dataset 2 and Supplementary Fig. 4 in Supplementary Information). Genes *cblA* and *cblB* were around 2-fold less expressed in cobalamin limitation than in nitrogen limitation, whereas *mmcm* was expressed almost at the same level (Fig. 7G).

One hour after vitamin B_12_ adding in cobalamin-limited reactors, during repletion phase, the expression of methionine cycle genes decreased by a factor 2 to 4 (Fig. 7A–C) and that of *cblA* and *cblB* by a factor 2 (Fig. 7D,E). During subsequent nutrient depletion, their expression returned to that at steady-state (Fig. 7A–E; Supplementary Fig. 4). This pattern of expression reflects noticeably intracellular cobalamin rate contents (Fig. 6C), with methionine cycle genes overexpressed in B_12_-limited cells and repressed in B_12_-replete cells. Expression of *mmcm* seemed not to be affected by vitamin B_12_ spike (Fig. 7F; Supplementary Fig. 4).

One hour after nitrate spike in nitrogen-limited reactors, the expression of *sahH* showed a slight increase while the expression of *metH*, *metK*, *cblA*, *cblB* and *mmcm* did not seemed to be affected by nitrogen addition (see Supplementary Fig. 4 in Supplementary Information). These genes did not show clear changes of expression in the depletion phase (Supplementary Fig. 4 in Supplementary Information). A decoupling between nitrogen status and the decrease in gene expression could explain the absence of regulation 24 hours after nitrogen spike. Overall, methionine cycle genes showed a clear trend directly related to vitamin B_12_ quota, and did not respond in an evident way to nitrates spike. Genes encoding accessory proteins CBLA and CBLB also showed an explicit pattern of expression induced by cobalamin quota but were not influenced by nitrogen status. Gene *mmcm* did not show any clear regulation of expression during the experiment with its expression level being almost the same for all chemostats independently of the cultures physiological state.

## Discussion

Haptophytes microalgae play an important role in carbon^17,18^ and sulphur cycling^20^ but their ability to respond to cobalamin variations is poorly known, despite the previously described impact of B_12_ limitation on phytoplankton growth and community composition^2,3,35^. The aim of this work was to (1) analyze B_12_ dependency of haptophytes based on molecular data and (2) provide insights into cobalamin molecular physiology of haptophytes by studying the model marine microalgae *Tisochrysis lutea*. This work combined bioinformatic searches, growth tests in batch and chemostat at different levels of B_12_ availability with the analyses of cobalamin quotas and expression of genes involved in B_12_ metabolism.

Nineteen haptophyte species across six orders and nine families were assessed for the presence of methionine synthase isoforms. All species investigated encoded the B_12_-dependent *metH* only. It has been observed that cobalamin auxotrophy is determined by the presence of *metH* and the absence of the cobalamin-independent methionine synthase *metE*^11,12^. Our results, mainly based on transcriptomic datasets, suggest that all of these haptophytes are cobalamin auxotrophs. No *metE* sequence of haptophytes was retrieved from the MATOU database, supporting the idea that species of this phylum are B_12_-requiring for growth. On the other hand, Croft *et al*.^11^ reported the occurrence of 8 haptophyte species among the 22 analyzed that did not require cobalamin^11^. It must be pointed out that 2 species over 22 were grown in their study and the remaining 20 were compiled from literature without any information about bacterial contamination. Recently, Helliwell *et al*. (2011) demonstrated the role of some bacteria so tightly attached to calcifying and non calcifying cells of *E*. *huxleyi* that they could not be disrupted with antibiotics, potentially providing the microalgae with vitamin B_12_^12^. Among 8 species considered as B_12_-independent 6 belong to Coccolithales. Strongly-attached, antibiotic-resilient bacteria may have been a B_12_ source for these species. The present paper is the first attempt to compile existing information based on molecular analyses for this phylum and, as no study found a species of Haptophyta phylum encoding *metE* nor a pseudogene, we assume that haptophytes are in majority cobalamin-dependent. This would be the first microalgae phylum gathering exclusively cobalamin auxotrophs, suggesting that their common ancestor did not encode *metE*, while in other phyla only certain species would have lost the B_12_-independent methionine synthase.

In order to explain the B_12_ molecular physiology of haptophytes, we selected the model species *T*. *lutea* for which comprehensive genomic and transcriptomic data were made available. Growth assays in natural seawater without cobalamin enrichment confirmed results of *in silico* approach as *T*. *lutea* was B_12_-limited two days after inoculation. Moreover, the absence of calcified coccoliths on the cells prevented presence of non detectable bacteria after strain purification. Adding methionine instead of B_12_ allowed *T*. *lutea* to develop, showing its ability to uptake and assimilate dissolved methionine to make up for cobalamin deprivation. This is consistent with another study^11^ demonstrating that the B_12_-dependent freshwater chlorophyte *Lobomonas rostrata* could be grown for several subcultures with METH products (*i*.*e*. methionine and folic acid). Our control without B_12_ exhibited 10 times lower maximal biomass compared with the control grown with 40 ng L^−1^ (24 pmol L^−1^) B_12_, suggesting around 4 ng L^−1^ (2.4 pmol L^−1^) cobalamin concentration in seawater. This is consistent with what was observed by Panzeca *et al*.^36^ and Suffridge *et al*.^35^ who estimated cobalamin concentrations ranging from 0.2 to 4 pmol L^−1^ in open oceans and 11 to 15 pmol L^−1^ in coastal ecosystems^35,36^ and indicating that vitamin B_12_ in natural seawater is limiting for this species. The maximal biomass obtained when *T*. *lutea* was grown with 500 g L^−1^ methionine was almost 3 times higher than the negative control but the concentration tested here was 625 times the maximal concentration found in seawater, that ranged from 0.27 ng L^−1^ offshore to 790 ng L^−1^ near the coast^37^. Recently, Suffridge *et al*.^35^ reported particulate methionine concentrations in seawater along a Mediterranean transect ranging from 0.30 to 3 ng L^−1^ ^35^. These findings mean that methionine concentrations in natural environment are likely to be limiting for *T*. *lutea* development and support the idea that auxotrophic microalgae need to be supplemented with a readily available cobalamin source such as vitamin-producing bacteria^14^, cell lysate or B_12_-remodeling algae, that are able to convert the less bioavailable pseudocobalamin into a readily accessible vitamin B_12_ form^38^.

We investigated the molecular physiology of *T*. *lutea* in batch and chemostat by focusing on the expression dynamics of genes involved in vitamin B_12_ use, transport and conversion. Genes *cblA* and *cblB*, encoding proteins transporting cobalamin to the mitochondrion, were down-regulated under B_12_ starvation in batch and B_12_ limitation in chemostat compared with the N-limited controls. This suggests that when B_12_ is limiting, cobalamin-dependent activities in the mitochondrion are reduced, possibly in favor of other cellular processes. Methionine cycle genes *metH*, *metK* and *sahH* and B_12_-dependent *mmcm* were not clearly affected by cobalamin starvation in batch. This differs from the results of Bertrand *et al*.^25,27^ for the B_12_-requiring diatom *Thalassiosira pseudonana*, which exhibited an overexpression of methionine cycle genes in cobalamin starvation with respect to replete conditions^25,27^. As expression pattern in batch experiments could be the result of numerous cellular processes related to the absence of cell division, these results must be viewed with caution. To bypass this, we implemented cultures in chemostat.

In this experiment, methionine cycle genes, *cblA* and *cblB* exhibited dynamics remarkably mirroring cobalamin quotas, with an overexpression in B_12_ limitation and downregulation in B_12_ repletion. These results could mean that upregulation of *metH*, *metK* and *sahH* is needed in cobalamin-limited environments to maintain optimal biochemical kinetics for methionine production and SAM cycling. Interestingly, expression of *mmcm*, that catalyzes the conversion of succinyl-CoA to methylmalonyl-CoA in the mitochondrion with cobalamin as cofactor, remained identical independently of vitamin limitation in batch and chemostat. It has been suggested that B_12_-dependent MMCM is not vital for growth as not all cobalamin-requiring microalgae possess it^12^. Also, the reaction of MMCM is one of many entries in TCA cycle and there could be other mechanisms of regulation at this metabolic level which could explain the lack of modifications in *mmcm* expression. In the proteomic dataset from the N-limited chemostat described by Garnier *et al*.^39^, methionine cycle proteins and MMCM belonged to the top 400 highest accumulated proteins over the 4330 identified during steady-state. Proteins CBLA and CBLB were not detected in their experiment^39^. In general, our expression analysis trends are in accordance with their proteomic results, as genes *cblA* and *cblB* were the lowest expressed.

To our knowledge, this is the first time that an analysis of B_12_ molecular physiology of a microalgae has been conducted in chemostat with accurately described nutrient states. More notably, this is the first time that expression dynamics of methionine cycle genes and B_12_ transporters to the mitochondrion are correlated to slight changes in vitamin B_12_ status in a marine microalgae, with rapid response no later than one hour after nutrient amendment. This fast regulation has been reported for *T*. *lutea* genes coding for nitrate and nitrite transporters (*TlNrt2*.*1* and *TlNrt2*.*3*) after addition of different nitrogen substrates^40^. This suggests that haptophytes hold quick acclimation mechanisms to nutrient availability that might explain their ecological success. The fact that these three methionine cycle genes, although not all coding for B_12_-dependent enzymes, are regulated in the same way raises the question of a common regulation system. Transcription factors are among major players in regulating gene expression, and some of them have already been described for *T*. *lutea* and related to oxidative stress response, triacylglycerol synthesis and photosynthesis^41^. Genes *cblA* and *cblB* were found to belong to a same group regulated by a shared transcription factor but methionine cycle genes were not gathered in a same module^41^. McRose *et al*.^42^ identified riboswitches affiliated with genes overexpressed in thiamine (vitamin B_1_) starvation in haptophytes microalgae^42^. Therefore, it is likely that such regulation mechanism would play a role in regulating, directly or not, cobalamin-related genes.

In conclusion, this is the first time that B_12_ dependency of haptophytes has been investigated. Based on 19 species surveyed, and since no haptophyte from the MATOU database was found encoding cobalamin-independent methionine synthase, we propose that haptophytes are cobalamin auxotrophs. Independence from vitamin B_12_ has been described as a mosaic pattern across evolution^11–13^ where Haptophyta would be the first microalgae phylum to gather only cobalamin-dependent species. The analysis of B_12_ molecular physiology of the model haptophyte species *T*. *lutea* has been undertaken. A controlled approach using chemostat cultures was performed to define precisely ecophysiological states, demonstrating the common assertion that this type of approach is of great interest when analyzing fine and rapid molecular changes in microorganisms^43,44^. Based on these results, we propose that haptophytes use different strategies to make up for cobalamin deprivation that include methionine assimilation, short-term regulation mechanisms in case of sudden B_12_ supply, such as cobalamin-producing bacteria excretion or cell lysis, and a preferential B_12_ allocation in the methionine cycle for METH activity. These results point out the importance of this cofactor in haptophytes cellular processes and represent a first attempt to understand the response of these ecologically important communities in vitamin B_12_-limited environments.

## Methods

### Sequence similarity search and validation

*In silico* analyses were realized by TBlastN and BlastP sequence similarity searches of the proteins on the new *T*. *lutea* genome^31,33^ with following entries (Uniprot): *Chlamydomonas reinhardtii* METH (A8HYR2) and METE (A8JH37), *E*. *huxleyi* METH (R1CGJ7), MAT from *Escherichia coli* (P0A817) and *Arabidopsis thaliana* (Q9SJL8), *Homo sapiens* and *A*. *thaliana* SAHH (P23526; O23255), *Rattus norvegicus* CBLA (D3ZNY3), *Homo sapiens* CBLB (Q96EY8) and MMCM (P22033), *Propionibacterium freudenreichii* subsp. *shermanii* MMCM (P11653). Homologous genes identified this way were searched again in *T*. *lutea* genome using TBlastX (expected threshold 1E-1). Conserved functional domains were identified by alignment of nucleic and proteic sequences in NCBI (METH: PFAM02574; METK: PFAM02773; SAHH: PFAM05221; CBLB: PFAM01923; MMCM: PFAM01642, PFAM02310 and PFAM08497).

Searches for methionine synthase isoforms in other haptophytes were firstly realized by BlastX of *T*. *lutea* METH on NCBI database, allowing to retrieve METH from *Chrysochromulina* sp. CCMP 291, *Thalassiosira pseudonana* CCMP 1335 and *Phaeodactylum tricornutum* CCAP 1055/1. The iMicrobe database was then queried, yielding sixteen haptophyte transcriptomes samples corresponding to 9 haptophyte families (see Table 1 in Supplementary Material for sequence references). TblastN of METE from *C*. *reinhardtii* (XP_001702934.1, NCBI) and *P*. *tricornutum* (B7G1X4, Uniprot); and METH from *T*. *lutea* were realized on the transcriptomes. Transcripts were translated into proteins using NCBI ORFfinder. Protein sequences were then aligned with METH from *T*. *lutea* and *T*. *pseudonana* and METE of *P*. *tricornutum*. Protein sequences were also verified by sequences alignments and conserved domains analyses. Identity and similarity with *T*. *lutea* sequences were estimated with LALIGN tool. Sequences alignment was conducted with MUSCLE (full mode) on the 21 METH sequences with *T*. *pseudonana* and *P*. *tricornutum* taken as outgroup. On total, 1206 positions were conserved on the 1508 initial (80%). Curation step was done with Gblocks tool, allowing gap positions within the final blocks and phylogenetic tree was realized with PhyML (100 bootstraps). Sequences homologous to genomic and proteic METE from *P*. *tricornutum* and *C*. *reinhardtii* were searched in the Marine Atlas of Tara Ocean Unigenes (MATOU) for eukaryotic data^32^ using BlastP and TBlastN (expected threshold 1E-1).

### Algal strain and purification

To limit bacterial contamination, a purification step was carried out on *Tisochrysis lutea* CCAP 927/14 strain with an antibiotic treatment mix prepared following the method described by Cho *et al*.^45^. For the following experiments, an inoculum from purified *T*. *lutea* culture was transferred three times every 10 days. Ten percent volume were transfered each time in new Erlenmeyer flasks containing Conway medium enriched sterile seawater^46^ with B_12_ omitted in order for the cells to progressively run out their B_12_ quota. Axenicity was verified in all the experiments by epifluorescence microscopy and cytometric analysis using SYBR™ Green staining (Lonza, USA) and by plating on Marine Agar (BD Difco™, Becton Dickinson Company, USA). Petri dishes were then incubated 3 days at 25 °C before further observation. When no bacteria or colony were observed, strains were considered axenic.

### Microalgal cultures

#### Microtiter plate growth assay

A growth assay was carried out to assess *T*. *lutea*’s cobalamin requirement and to investigate whether the microalgae can be grown with methionine, end product of METH activity. Two milliliters inoculum from the last cobalamin-limited batch were dispatched in test tubes (final concentration 1 10^6^ cells mL^−1^) and enriched with Conway medium either containing 40 ng L^−1^ B_12_, or cobalamin free or cobalamin free enriched with 0.5 mg L^−1^ L-methionine (HPLC grade Sigma; >99% purity). Six replicates were inoculated for each condition in a microtiter plate that was incubated at 26 ± 1 °C and 90 μmol m^−2^ s^−1^. O.D.680 was monitored by spectrophotometry (Quant, BIO-TEK Instruments inc, USA).

#### Batch experiment

To identify modifications in *T*. *lutea*’s molecular physiology during vitamin B_12_ consumption, a cobalamin-limited batch experiment was first performed. Three 1-liter autoclaved glass bottles were inoculated at 2.5 10^6^ cells mL^−1^ and enriched with Conway medium with 40 ng L^−1^ B_12_. Cultures were homogenized by filtered air bubbling (Midisart 0.2 m, Sartorius) and were placed at 27 ± 1 °C with a continuous irradiance of 180 μmol m^−2^ s^−1^ photons. Cellular concentration was followed by counting Lugol stained cells with Malassez haemocytometer. Samples for quantitative analysis of B_12_ and qPCR were taken at days 2 (early exponential phase) and 20 (stationary phase).

#### Chemostat experiment

Two inoculi were acclimatized at 27 ± 1 °C under a continuous irradiance of 180 μmol m^−2^ s^−1^ photons. After 10 days, they were divided into four autoclaved glass bottles filled with 4.5 L sterile seawater enriched with modified Conway medium, with a final concentration of either 40 ng L^−1^ B_12_ or 25 mg L^−1^ NaNO_3_ to ensure limitation in B_12_ or nitrogen (N) respectively. Bottles were set up in chemostat supplied with a continuous input of the media described above. The experiment was carried out in duplicate for each condition at 27 ± 1 °C with a continuous irradiance of 400 μmol m^−2^ s^−1^ photons and pH maintained at 8.2 by CO_2_ enrichment. Cultures were homogenized properly with a constant input of filtered air and a magnetic stirrer. Dilution rate (D) was adjusted at 0.5 d^−1^ and monitored daily by weighing system output. Populations were monitored by particulate carbon and nitrogen measurement and cell counting in Malassez haemocytometer. To confirm adequate nutrient limitation and to study physiological modifications after nutrient adding, discreet nutrient input was done at day 25 either with 25 g L^−1^ NaNO_3_ or 40 ng L^−1^ B_12_ for the N-limited and B_12_-limited chemostats respectively.

### Biochemical analyses

#### Particulate carbon and nitrogen

Particulate organic nitrogen and carbon were measured by filtering 20 10^6^ cells on 25 mm precombusted GF/F microfibers filters (0.7 m, Whatman, UK). Filters were then dried at 65 °C for at least 12 hours. Particulate organic nitrogen and carbon were analyzed with a CN elemental analyzer (Flash 2000, Thermo Fisher Scientific, Waltham, USA).

#### B_12_ measurements

Intracellular B_12_ quantification was assessed with an ELISA test kit (Immunolab, Germany) with a sensitivity of 0.3 ng mL^−1^. Zhu *et al*. showed that neither salinity nor dissolved organic matter do interfere with test quality^47^. This procedure allowed to measure the different chemical forms of vitamin B_12_ (cyanocobalamin, methylcobalamin, adenosylcobalamin and hydroxycobalamin) with a cross-reactivity of 98–100% among the chemical variants^47^. Cell pellets (80–150 10^6^ cells) were resuspended in 100 μL PBS buffer (provided in the kit) and extraction was undertaken by boiling 15 minutes at 99 °C as previously described for microalgae^48^. Supernatant was collected after centrifugation (16 000 *g*, 5 minutes, 4 °C). Extracts were assayed following the method given in the kit, which provided cyanocobalamin solutions as standards. Absorbences at 450 and 620 nm (three technical replicates) were measured with a spectrophotometer (μQuant, BIO-TEK Instruments inc, USA). Standards and samples absorbency was defined as follows: O.D.450–O.D.620. Cobalamin concentration of samples was calculated using the calibration curve equation.

### RNA extraction and RT-qPCR

Cell lysis was obtained by adding 1 ml Trizol (Life Technologies) and 200 μL chloroform to cell pellets of 300 10^6^ cells. Samples were then purified with RNeasy™kit (Qiagen) following the provided protocol. RNA purity and concentration were verified using a spectrophotometer (Infinite 200 PRO) at 260 and 280 nm. Diluted samples of 250 ng μL^−1^ were treated with DNase (Promega) 1 hour at 37 °C. Reverse transcription was performed using High-Capacity cDNA Reverse Transcription kit (Applied Biosystems) according to the provided protocol. Primer efficiency was quantified following protocol of Schmittgen *et al*.^49^ (see Table 2 on Supplementary Information for primers sequences). Primer specificity was estimated with a denaturation cycle at PCR end. PowerUp™SYBR™Green mix (Applied Biosystems) was used for RT-qPCR. Thermocycler (Mx3000P, Agilent) parameters were set as follows: 1 cycle of 15 minutes at 95 °C, 40 cycles of 30 seconds at 95 °C and 30 seconds at 60 °C. Six genes coding for 18S, actin, EF1, GAPDH, tubulin and ubiquitin were tested as housekeeping genes. As GAPDH exhibited low cycle threshold (Ct) variations with the same order of magnitude than target genes it has been selected as reference gene (see Fig. 1 of Supplementary Information). Gene expression was calculated by raising negative cycle threshold values of each pair of primers and dividing it by mean expression of reference gene. Raw data of genes expression is available in Supplementary Datasets 1 and 2 for the batch and chemostat experiments respectively.

## Supplementary information

Dataset_1

Dataset_2

Supplementary_Information_Nef_et-al_scientific_reports
