# Supplementary material for: How haptophytes microalgae mitigate vitamin B12 limitation
Source: Sci Rep. 2019 Jun 10;9:8417. doi: 10.1038/s41598-019-44797-w (PMC6557843; doi:10.1038/s41598-019-44797-w)
Supplement: Supplementary file 3 — Supplementary_Information_Nef_et-al_scientific_reports [file 41598_2019_44797_MOESM3_ESM.docx]

**Supplementary Information**

**How haptophytes microalgae mitigate vitamin B_12_ limitation**

**Charlotte Nef*, Sébastien Jung, Francis Mairet, Raymond Kaas, Dominique Grizeau and Matthieu Garnier**

*Corresponding author

**Supplementary Table 1.** Reference of cobalamin-dependent methionine synthase (*metH*) sequences compiled for phylogenetic analysis. Identity and similarity percentages with *T. lutea* sequence are provided.

| **Data** | **Family** | **Species** | **Strain** | **iMicrobe sample reference** | **iMicrobe sample collection site** | **Sequence name** | **Evalue** | **Score** | **Identity/Similarity (%)** |
| --- | --- | --- | --- | --- | --- | --- | --- | --- | --- |
| Genomic | Isochrysidaceae | *Tisochrysis lutea** | CCAP 927/14 |  |  | 1602 | - | - | - |
| Transcriptomic | Isochrysidaceae | *Isochrysis* sp. | CCMP 1244 | MMETSP1090 | North Atlantic | CAMNT_0042019453 | 0 | 2407 | 86.8/95.8 |
| Transcriptomic | Isochrysidaceae | *Isochrysis* sp. | CCMP 1323 | MMETSP0944 | Irish Sea | CAMNT_0021684687 | 0 | 3001 | 92.7/97.9 |
| Transcriptomic | Prymnesiaceae | *Chrysochromulina rotalis* | UIO 044 | MMETSP0287 | North Sea | CAMNT_0003299591 | 0 | 2098 | 76.2/88.9 |
| Transcriptomic | Prymnesiaceae | *Chrysochromulina brevifilum* | UTEX LB 985 | MMETSP1094 | English Channel | CAMNT_0015937587 | 0 | 2052 | 74.9/89.5 |
| Transcriptomic | Prymnesiaceae | *Prymnesium parvum* | Texoma1 | MMETSP0006 | Lake Texoma, Oklahoma | CAMNT_0024934099 | 10^-103^ | 379 | 77.7/91.0 |
| Transcriptomic | Pavlovophyceae | *Pavlova gyrans* | CCMP 608 | MMETSP1466 | Helford river, Falmouth, UK | CAMNT_0053406913 | 0 | 1917 | 70.9/87.2 |
| Transcriptomic | Pavlovophyceae | *Pavlova lutheri* | RCC 1537 | MMETSP1463 | North Sea | CAMNT_0053336917 | 0 | 1897 | 71.8/86.6 |
| Transcriptomic | Phaeocystaceae | *Phaeocystis antarctica* | CCMP 1374 | MMETSP1444 | Ross Sea | CAMNT_0043842521 | 10^-118^ | 424 | 77.1/89.4 |
| Transcriptomic | Noëlarhabdaceae | *Gephyrocapsa oceanica* | RCC 1303 | MMETSP1364 | - | CAMNT_0027971079 | 0 | 2387 | 86.7/95.7 |
| Transcriptomic | Noëlarhabdaceae | *Emiliania huxleyi* | 379 | MMETSP0994 | English Channel | CAMNT_0041613577 | 0 | 2407 | 86.8/95.8 |
| Transcriptomic | Noëlarhabdaceae | *Emiliania huxleyi* | PLY M219 | MMETSP1150 | - | CAMNT_0023937657 | 0 | 2409 | 86.8/95.7 |
| Transcriptomic | Noëlarhabdaceae | *Emiliania huxleyi* | CCMP 370 | MMETSP1154 | - | CAMNT_0024259279 | 0 | 2408 | 86.8/95.7 |
| Transcriptomic | Calcidiscaceae | *Calcidiscus leptoporus* | RCC 1130 | MMETSP1334 | South Atlantic | CAMNT_0007365605 | 2*10^-98^ | 361 | 77.4/90.9 |
| Transcriptomic | Pontosphaeraceae | *Scyphosphaera apsteinii* | RCC 1455 | MMETSP1333 | Spanish coast, Mediterranean Sea | CAMNT_0007313019 | 6*10^-86^ | 319 | 79.6/91.8 |
| Transcriptomic | Pleurochrysidaceae | *Pleurochrysis carterae* | CCMP 645 | MMETSP1136 | - | CAMNT_0010116929 | 10^-145^ | 517 | 78.9/91.0 |
| Transcriptomic | Coccolithaceae | *Coccolithus pelagicus* ssp. *braarudi* | PLY 182g | MMETSP0164 | English Channel | CAMNT_0025532985 | 3*10^-90^ | 333 | 76.5/89.5 |
| Proteomic | Prymnesiaceae | *Chrysochromulina* sp. | CCMP 291 | - |  | KOO28302.1 | 0 | 2006 | 74.9/88.4 |
| Proteomic | Noëlarhabdaceae | *Emiliania huxleyi* | CCMP 1516 | - |  | XP_005791493.1 | 0 | 2228 | 82.0/91.4 |
| Proteomic | Thalassiosiraceae | *Thalassiosira pseudonana* | CCMP 1335 | - |  | XP_002293120.1 | 0 | 1352 | 55.8/79.9 |
| Proteomic | Phaeodactylaceae | *Phaeodactylum tricornutum* | CCAP 1055/1 | - |  | XP_002184461.1 | 0 | 1320 | 56.3/78.7 |

*See Carrier et al. (2018) and Berthelier et al. (2018) for *T. lutea* genome.

**Supplementary Table 2.** Primer sequences used for RT-qPCR analyses.

| **Gene** | **Primer name** | **Primer sequence (5’ → 3’)** |
| --- | --- | --- |
| *metH* | METH-F | CGAACTGCTCCATCACAAGA |
|  | METH-R | GACGGATGCCTTGGTACTTG |
| *metK* | METK-F | TATTGCCGTTCTTCAGCTTG |
|  | METK-R | AAGTACACTGGCCCGGAGAT |
| *sahH* | SAHH-F | GCTGGAGGAGTACTGGTGGT |
|  | SAHH-R | CTCATGGAGGAGGAGGGTTG |
| *cblA* | CBLA-F | GTGCAGCTTGTCCAGCACT |
|  | CBLA-R | CTCAGTCATGTGACCCAACG |
| *cblB* | CBLB-F | CGTCGATCCATCCACCAC |
|  | CBLB-R | TGAGCCAACATCAAGTAGTCG |
| *mmcm* | MMCM-F | CACGCAGTCTGTCGCAAG |
|  | MMCM-R | CGAGAAGACCAAGGGAGACT |


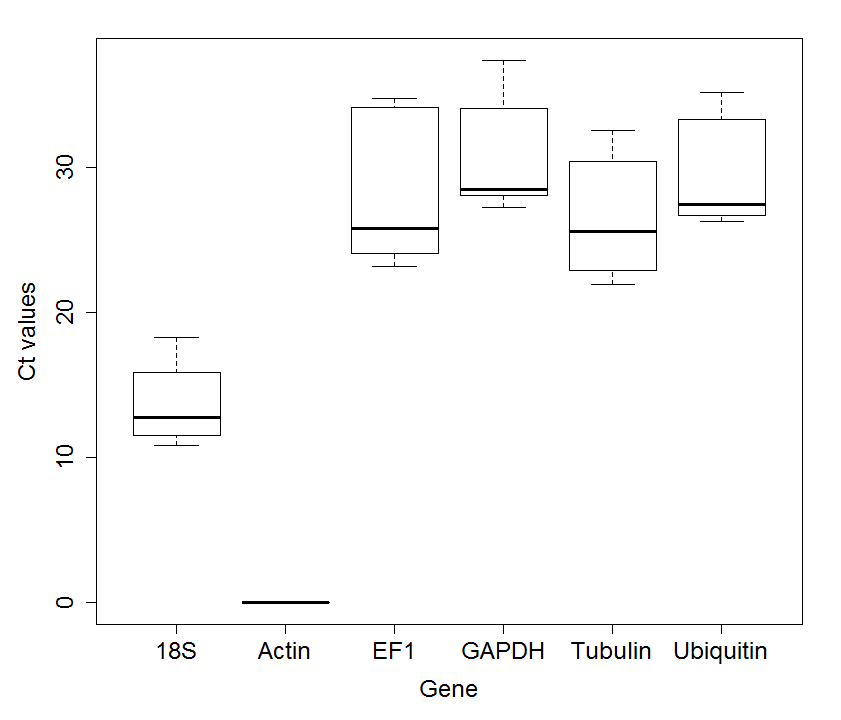


**Supplementary Figure 1.** Boxplot of Ct variation for reference genes tested with bold line indicating median (n = 18).


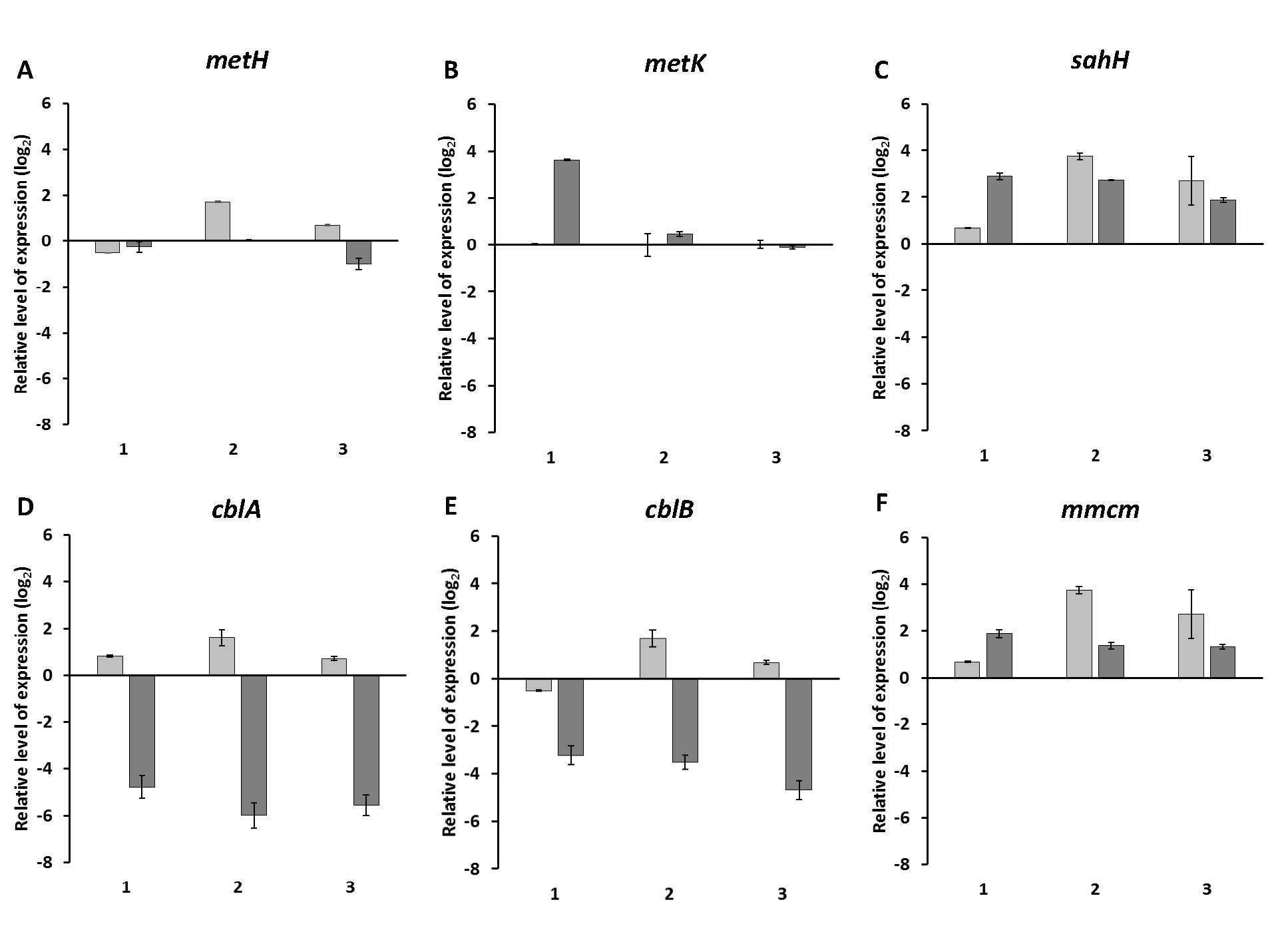


**Supplementary Figure 2.** Genes expression in B_12_-limited batch cultures of *T. lutea*. Relative levels of expression of (A) *METH*, (B) *METK*, (C) *SAHH*, (D) *CBLA*, (E) *CBLB* and (F) *MMCM* genes. Values represent mean expression level at exponential phase (gray) and stationary phase (black) quantified and normalized using *GAPDH* as reference gene. Data are log_2_ normalized. Values are shown for each biological replicate (1, 2 and 3). Bars indicate means of technical triplicate measurements and error bars represent one standard deviation.


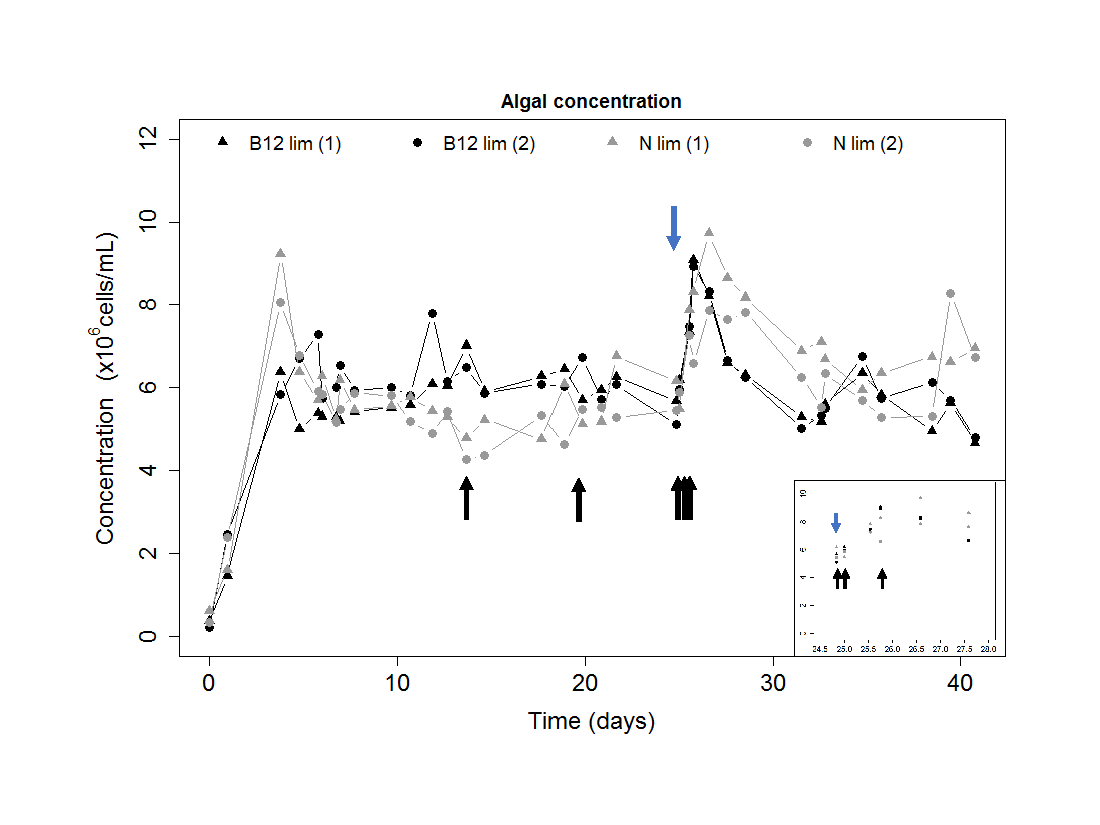


**Supplementary Figure 3.** Algal concentration for chemostat cultures of *Tisochrysis lutea*. Blue arrows represent nutrient spike, black arrows indicate sampling points (Ss 1, 2, 3, spike + 1 h and spike + 24 h). Data are for B_12_-limited (black) and nitrogen-limited (gray) chemostats.


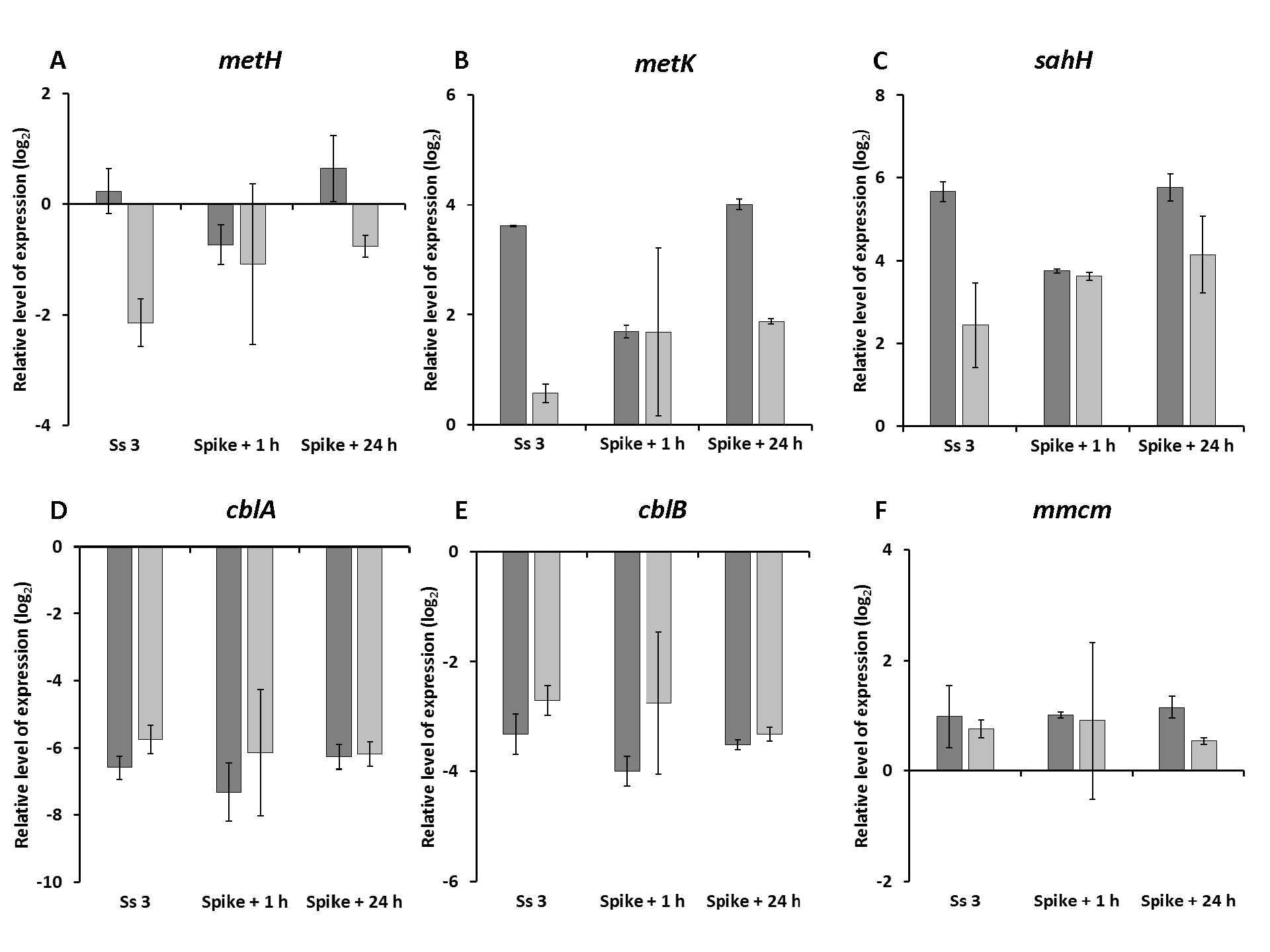


**Supplementary Figure 4.** Genes expression in chemostat cultures of *T. lutea* during steady-state (Ss), 1 and 24 hours after nutrient spike for (A) *METH*, (B) *METK*, (C) *SAHH*, (D) *CBLA*, (E) *CBLB* and (F) *MMCM* genes. Data are for B_12_-limited (black) and nitrogen-limited (gray) chemostats and are log_2_ normalized. Values represent mean expression level quantified and normalized using *GAPDH* as reference gene. Bars indicate means of biological duplicates and error bars represent the range.

**References**

Carrier, G. C. *et al.* Draft genome and phenotypic characterization of *Tisochrysis lutea* strains. Toward the production of domesticated strains with high added value. *Algal Res*. **29**, 1–11 (2018).

Berthelier, J. *et al.* A transposable element annotation pipeline and expression analysis reveal potentially active elements in the microalgae *Tisochrysis lutea*. *BMC Genomics* **19** (2018).
